# 

**Published:** 1894-10

**Authors:** 


### CONTENTS—OCTOBER, 1894.—VOL. X., NO. 4.

ORIGINAL COMMUNICATIONS—

Two Cases of Post-partum Hemorrhage. A. S. Fuller, M. D., Houston 147
The Importance of Early Recognition of Tuberculosis—Its Curability with Special Reference to Climatology. T. W. Conerly, M. D., San Angelo . . . . . 151
Report of a Case of Abdominal Hysterectomy. J. Cummings, M. D., Austin . . . . . 157
An Answer to Dr. F. T. Oates on Treatment of Malarial Hæmatinuria. W. W. Walker, M. D., Schulenburg . . . . . 161
A Case of Spina-Bifida—Operation. J. T. Feild, Fort Worth . . . . . 163

CURRENT MEDICAL LITERATURE—

Department of Practice of Medicine. Prof. A. J. Smith, M. D. . . . 165
Gynecological Notes. T. Flavin, M. D., Galveston . . . . . 171
Notes on Dermatology. Isadore Dyer, M. D., New Orleans . . . . . 173

CORRESPONDENCE—

Typhoid Fever. Letter from Dr. Rogers . . . . . 174

SOCIETY NOTES—

Northwest Texas Medical Association . . . . . 176

EDITORIAL—

Texas Medical Association . . . . . 177
Our Contributors . . . . . 181
What Next? . . . . . 182
"Railway Spine" . . . . . 184

MEDICAL NEWS AND MISCELLANY—

Numerous Paragraphs . . . . . 184
As Others See Us . . . . . 192
A Great Sanitarian on Spinal Concussion . . . . . 193

BOOK NOTICES— . . . . . 195
PUBLISHERS' NOTES . . . . . 200

